# When more isn’t better: evidence for an instructional equivalence hypothesis in multimedia design

**DOI:** 10.3389/fpsyg.2025.1718397

**Published:** 2025-11-20

**Authors:** Katie J. Schmidt, Kristie R. Dukewich, C. Itzel Symonds, Alex V. Thrasher

**Affiliations:** Department of Psychology, Kwantlen Polytechnic University, Surrey, BC, Canada

**Keywords:** pedagogy, multimedia, educational materials, computer learning, cognition

## Abstract

Pedagogical theories suggest that effective multimedia can reduce extraneous cognitive load and help students create mental models of new learning. Theoretically derived and empirically supported design principles are widely assumed to improve learning outcomes, but most of the principles have been studied in relative isolation. This study was conducted as a strong test of multimedia design for learning controlling for content and pedagogy. We presented participants with short educational videos using three different multimedia formats: Rich multimedia, sparse multimedia, and no multimedia. Despite the strong theoretical and empirical foundations for this experiment, there was no significant effect of multimedia design on learning outcomes, *F*(2, 126) = 0.52, *p* = 0.60, η_p_^2^ = 0.008. Need for Cognition scores were measured and included as a covariate; however, they did not significantly predict performance across conditions, *F*(1, 63) = 0.25, *p* = 0.62, η_p_^2^ = 0.004. Contrary to expectation, multimedia design had no measurable impact on student learning. To account for this pattern, we introduce the *Instructional Equivalence Hypothesis*—the proposal that when content and pedagogy are effective and internally aligned, the format of multimedia presentation may be functionally interchangeable. This framework challenges a central assumption of the multimedia learning literature and invites a reevaluation of how design principles are theorized, tested, and applied in educational settings.

## Introduction

Multimedia design for learning refers to the intentional design of multiple forms of media—such as text, images, narration, animation, and video—to support conceptual understanding of course content (Mayer, 2001). The goal is not just to make learning materials aesthetically appealing, but to enhance comprehension and learning. Good multimedia design is meant to help learners manage their cognitive resources and organize information into new or existing knowledge structures, facilitating conceptual understanding and long-term retention.

The research on multimedia design for learning is strongly grounded in cognitive theories of how people learn. Sweller’s Cognitive Load Theory (CLT) suggests that learning is optimized when a learner’s cognitive load is managed to minimize unnecessary demands on working memory, allowing more cognitive resources to be devoted to processing and understanding essential instructional content (Sweller et al., 2019). The theory implies that instructional design should aim to reduce extraneous demands on cognitive load, manage the intrinsic difficulty of the material, and optimize cognitive load to facilitate effective learning and schema construction. The Cognitive Theory of Multimedia Learning (CTML) is an applied theory of multimedia design that integrates CLT and Baddeley’s working model of memory (Baddeley and Hitch, 1974). CTML advocates for a division of labor between modalities: auditory channels should convey verbal material, while visual channels are best used for illustrative content such as figures or animations (Mayer, 2001). By using working memory resources efficiently, CTML suggests that learners will have sufficient cognitive load to help them either connect new learning to prior knowledge or develop new mental models. Finally, the Cognitive-Affective Theory of Learning with Media (CATLM) proposes that multimedia design should support both cognitive processing and positive emotional engagement, as emotions can enhance motivation and deepen learning (see Mutlu-Bayraktar, 2024 for a review).

### Multimedia design for learning

A rich literature of empirical research investigating multimedia design for learning has identified at least 15 different design principles (Mayer, 2001). These principles provide evidence-based guidelines for educators and instructional designers to effectively manage and exploit cognitive load and/or emotional engagement to optimize learning (see Supplementary Table A for a list of the 13 most prominent principles from the literature).

The first, and most empirically supported, principle is the multimedia principle: people learn better from a combination of words and images rather than from words alone (Mayer and Anderson, 1992). Most of the remaining design principles provide guidance about how to combine words and images most effectively. For example, the modality principle suggests that learning is most effective when words are presented auditorily, saving the visual modality for processing of graphics and images (Mayer and Anderson, 1992).

Based largely on one-off learning experiences in the context of formal experiments, researchers have found that employing the principles for multimedia design for learning can have profound impacts on learning outcomes (Dukewich et al., 2025). For example, meta-analyses show that the modality principle—which recommends presenting words as spoken narration rather than on-screen text when paired with graphics—yields medium-to-large effect sizes, with one review reporting an average *g* ≈ 0.74 across dozens of experiments (Mayer, 2024).

Most published studies have focused on single multimedia-design principles—or at most pairs of principles—rather than examining how multiple principles interact with each other (Çeken and Taşkın, 2022). Moreover, studies can and often do confound content and pedagogy with multimedia design. Moreno and Mayer (2002) investigated the redundancy effect by presenting content on how the lightning process works. They explicitly describe the development of their materials such that the narration-only version was identical to the narration-visual animation version “with the exception that the animations had been deleted throughout the programs” (pp.158). This description implies that the content was originally designed to align with the multimedia presentation rather than designed to stand alone. That confound can be compounded in the measures of learning if test questions superficially aligned with the multimedia design. For example, learners who see a labeled diagram will have a benefit on a question that tests label recall. As a result, the questions used to evaluate learning may artificially favor multimedia conditions. This produces a methodological confound: the positive effects of multimedia might reflect an overlap between the test format, the content or pedagogical approach, and the multimedia design features rather than genuine learning.

### The current study

To determine whether multimedia effects persist when instructional quality is held constant, we conducted a strong test of the multimedia design principles and CTML, controlling for content and pedagogy. We developed scripts for three different content videos independently of the multimedia, and then developed three distinct multimedia conditions, rich multimedia (rich MM) that used all of the multimedia design principles for learning, sparse multimedia (sparse MM) that aligned with more traditional lecture slides, and no multimedia (no MM). We also piloted tested our knowledge questions to ensure they were sensitive enough to detect differences in learning if they were present. Based on the multimedia design principles and CTML, we hypothesized that learning outcomes would be superior in the rich MM condition compared to the spare MM and no MM conditions. We also hypothesized that participants would rate their subjective experience of the video higher when in the rich MM condition compared to sparse and no MM conditions.

While not our primary focus, we also measured participants’ Need for Cognition (NFC; Cacioppo et al., 1984) to capture individual differences in learners’ motivation to engage in and enjoy effortful cognitive activity. Prior research has shown that NFC is positively associated with academic achievement across diverse learning contexts (see Liu and Nesbit, 2024 for a meta-analysis), and that it can moderate the effectiveness of specific instructional features, including multimedia design elements (e.g., Kühl et al., 2014). Because NFC could potentially moderate the effects of our multimedia design manipulation, we included it as a covariate to control for the variance these individual differences might introduce.

## Method

### Pilot study

Four pilot studies were conducted to evaluate the relative difficulty and internal consistency of the knowledge tests associated with three educational videos on color, depth, and sound perception. Each video was approximately 10 min long and featured rich multimedia with audio narration. For full details of each pilot study, see Supplementary Table B.

In the first pilot (*N* = 16, 87.5% female, *M_age_* = 26.6), participants viewed videos on color and depth perception and completed 14 multiple-choice questions per topic (e.g., “Color serves as a signaling function. Which of the following is an example of this function?”). For the color video, the average was *M* = 6.75 and Cronbach’s alpha was *α* = 0.74, and for the depth video *M* = 6.67 and α = 0.75. Items with low predictive value were revised for difficulty (probability of correct) and predictive value (item-total correlation), and a second pilot (*N* = 30, 53.3% male, *M_age_* = 29.6) tested the revised items. Internal consistency improved (color: *M* = 7.13 and, *α* = 0.76; depth: *M* = 6.70 and *α* = 0.77). The four lowest-performing items per topic were removed, resulting in a 10-item test for each topic video.

A third pilot (*N* = 25, 80.8% female, *M_age_* = 24.9) introduced a new video on sound perception, followed by 14 test questions (*M* = 7.33, *α* = 0.67). After revising or removing low-performing items, a final pilot (*N* = 20, 80.0% female, *M_age_* = 23.1) confirmed improved reliability for the sound questions (*M* = 6.65, *α* = 0.75), and the test was reduced to 10 items.

### Participants

Participants were recruited online through Prolific, receiving £8.00 in compensation. Participants had to be at least 16 years of age.

An a-priori power analysis with G*Power found that 66 participants were needed to detect a small-to-medium effect size. After contending for any data that met exclusion criteria (i.e., the participant did not complete three or more questions), 65 participants remained (63.1% female, *M_age_* = 33.4). See Supplementary Table C for full demographic information.

### Materials and procedures

We conducted a within-subjects experiment with three conditions (rich MM, sparse MM, and no MM) and one measured covariate (NFC). The study design, hypotheses, and analysis plan were preregistered prior to data collection on the Open Science Framework (OSF; https://osf.io/8wf7y). This study was reviewed and approved by the Kwantlen Polytechnic University Research Ethics Board (REB), and all participants provided informed consent prior to participation.

#### Learning materials

The learning materials consisted of three 10-min-long videos. The videos were about color perception, depth perception, and sound perception. Each video included lecture material adapted from a university course on perception. Each script was written separately from the development of the multimedia that would accompany the narration. The narration for each video was recorded using OBS open software with the presenter in front of a greenscreen background to allow easy recombination of the audio track, presenter video, and background multimedia. We used CapCut to separate the audio narration from the greenscreen presenter video. The background multimedia was generated in PowerPoint and turned into a video file that was combined with the audio narration in CapCut. The videos were uploaded to YouTube for hosting.

**Figure 1:**
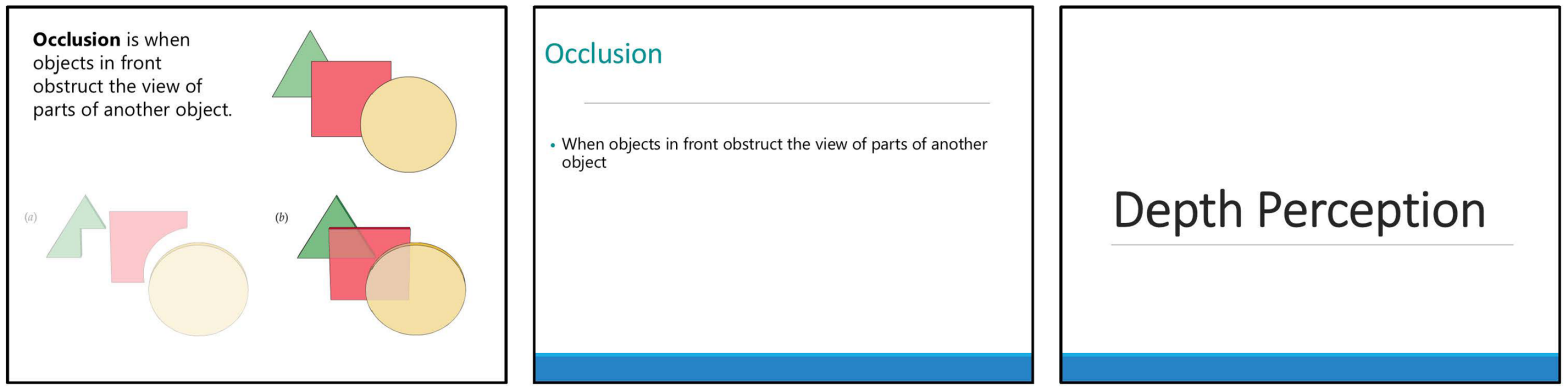
The multimedia conditions from left to right are: rich MM, sparse MM, no MM. These slides are taken from the depth perception video.

For each video, three versions were created: rich MM (see https://youtu.be/z4DCQfLqvBI?si=eN-2aP66f6qSt1No for an example), which used text and graphics organized as per the principles of multimedia design for learning; sparse MM (see https://youtu.be/sq7kr2TOCDo?si=d2vSCd7u3kDTIt1D for an example), which used bullet point text on a basic PowerPoint template with no images violating the multimedia principle, modality principle, redundancy principle, embodiment principle, and emotional design principle; and no MM (see https://youtu.be/UzOkP9KT3Bc?si=ZH_3ziAvss9NhJCj for an example), which used a static image of the title slide as a visual placeholder (see Figure 1).

#### Subjective ratings

The Lecture Engagement Questionnaire (LEQ; none *α* = 0.88; sparse *α* = 0.86; rich *α* = 0.78) was used as a measure of participant’s subjective ratings of the videos (Stull et al., 2018). It was a 6-point Likert scale ranging from 1 (*strongly disagree*) to 6 (*strongly agree*). It consisted of questions such as “I enjoyed learning this way” and “I would like to learn this way in the future.” See Supplementary material to view the entire questionnaire. The total engagement score consisted of the average of all the questions. Higher scores indicated more favorable subjective ratings (e.g., more engaged).

#### Need for cognition

NFC was measured using Cacioppo et al. (1984) Need for Cognition Scale (*α* = 0.78). The scale consisted of 18 questions such as “I prefer complex to simple problems” and “I find satisfaction in deliberating hard and for long hours.” The questions were rating on a 7-point Likert scale ranging from 1 (*totally disagree*) to 7 (*totally agree*). See Supplementary material for the entire questionnaire. The total NFC score consisted of the average of all the questions. Higher scores indicated higher NFC.

#### Learning performance

Learning performance was measured through a knowledge test, consisting of 30 multiple choice questions with 10 from each lecture (see Supplementary material for a list of all the questions). There were 14 factual questions and 16 transfer questions. Factual questions are questions that required recall or recognition of specific information explicitly presented in the learning material, while transfer questions are application-based questions that require answers that are not explicitly contained in the learning material (Fiorella et al., 2019). For example, a question regarding color perception was “How might altering the typical color of an object affect its identification in real-world scenarios?” The order of the questions and the order of the choices within each question were randomized. Answers were scored as one for correct and zero for incorrect. A final score was calculated using the sum of all the questions, ensuring that missed questions were counted as zero. Higher scores indicated better learning performance.

#### Time-on-task

Participants were also asked to indicate how much time they spend away from their screen during the experiment. They were asked if they walked away, opened another browser, or used another device during the experiment. All three questions were answered using an ordinal scale with the options 0 min, 1–5 min, 6–15 min, and 16+ minutes. The full questionnaire can be found in the Supplementary material.

##### Procedure

**Figure 2:**
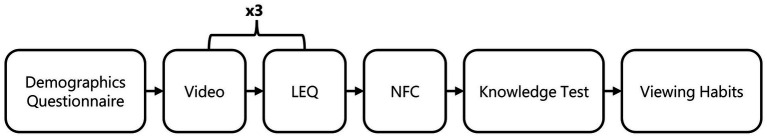
Each participant watched three videos, completing the LEQ after each individual video. The NFC, knowledge test, and time-on-task questionnaires were administered after all three videos and LEQs.

The online study was conducted through Qualtrics. After giving consent, participants completed a demographics questionnaire. Each participant saw three videos: one with rich MM, one with sparse MM, and one with no MM. Participants watched one video and then completed a LEQ regarding that video before proceeding to the next video. After the third video and LEQ, they answered the NFC scale and then were given a knowledge test consisting of questions about the videos.^1^ After the knowledge test, participants indicated their time-on-task. The study ended with a debriefing form and participants were compensated. See Figure 2 for a schematic representation of the procedure.

While an effort was made to equate the knowledge test questions for difficulty and reliability, there might have been some inherent intrinsic differences in the difficulty of the topics or in the participants’ overall familiarity with the content. To equate the difficulty of the conditions, the video topic for each multimedia condition was randomly assigned across participants. To avoid order effects, the order of the multimedia conditions was also randomized. See the Supplementary material for a matrix illustrating how the conditions were counterbalanced and randomized.

## Results

Two one-way ANCOVAs were conducted with multimedia condition (rich MM, sparse MM, no MM) as the within-subjects factor, NFC as the covariate, and both a knowledge test and LEQ as the dependent variables.

### Learning performance: knowledge test

**Figure 3:**
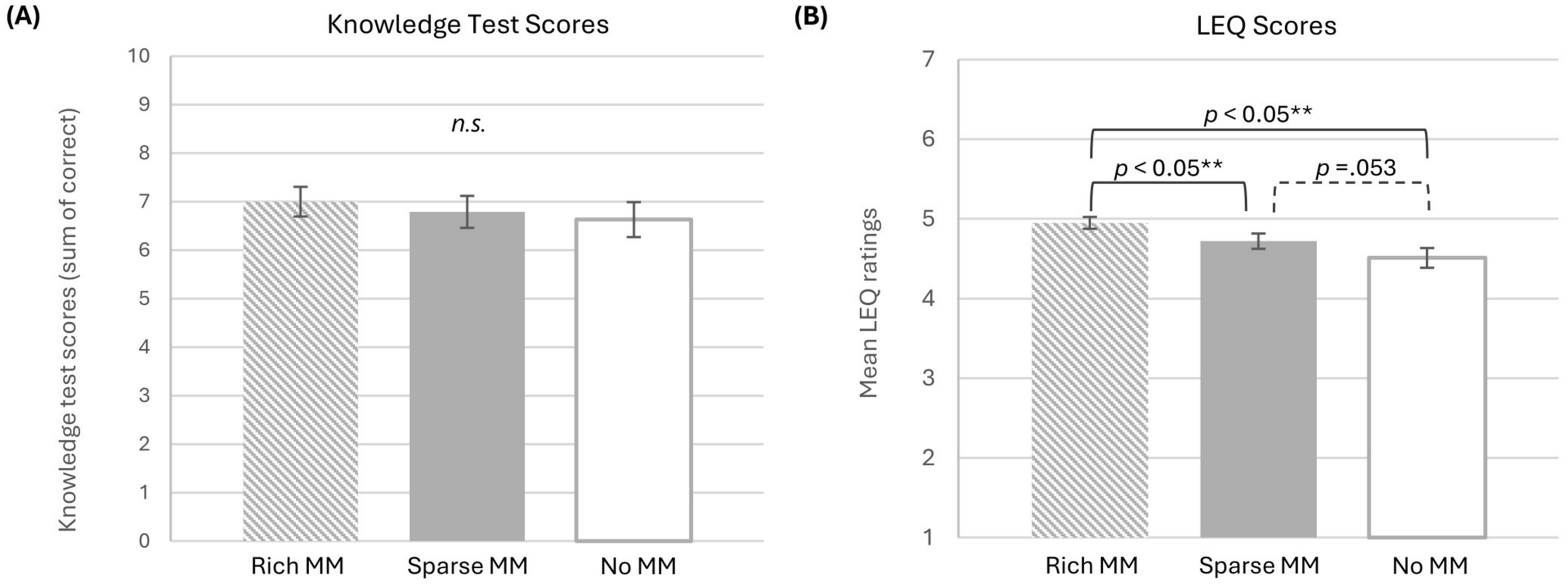
The average **(A)** knowledge test scores and **(B)** LEQ ratings for each dependent variable by instructor condition. Error bars represent ±1 standard error of the mean.

Means and standard deviations for each condition are presented in Table 1. Adjusted means from the ANCOVA are displayed in Figure 3A (for more details, see Supplementary Table D). Assumptions of independence, normality, and sphericity were met.

**Table 1:** Means and standard deviations for each condition and dependent variable.

Condition	Knowledge test scores		LEQ ratings	
	*M*	*SD*	*M*	*SD*
Rich MM	7.00	2.47	4.95	0.60
Sparse MM	6.78	2.64	4.72	0.78
No MM	6.63	2.88	4.50	1.02

On average, participants scored highest in the rich MM condition (*M* = 7.00, *SD* = 2.47), followed by the sparse MM condition (*M* = 6.78, *SD* = 2.64), and lowest in the no MM condition (*M* = 6.63, *SD* = 2.88).

The analysis revealed that NFC was not significant as a covariate, *F*(1, 63) = 0.25, *p* = 0.62, η_p_^2^ = 0.004, indicating NFC did not account for a significant proportion of variance in performance after controlling for multimedia condition. The main effect of multimedia condition was not significant, *F*(2, 126) = 0.52, *p* = 0.60, η_p_^2^ = 0.008.

A Bayesian repeated-measures ANOVA provided substantial evidence for the null hypothesis, indicating no effect of multimedia condition on learning outcomes. The null model had the highest posterior probability [P(M|data) = 0.588], and Bayes factors indicated the data were 5.57–12.31 times more likely under the null than under models including Multimedia or Multimedia × NFC effects (see Supplementary Table E).

### Subjective ratings: LEQ

Means and standard deviations for each condition are presented in Table 1. Adjusted means from the ANCOVA are displayed in Figure 3B (for more details, see Supplementary Table F). Assumptions of independence and normality were met. The assumption of sphericity was violated, so all *F-*values are reported using a Greenhouse–Geisser correction.

On average, participants reported the highest engagement in the rich MM condition (*M* = 4.95, *SD* = 0.60), followed by the sparse MM condition (*M* = 4.72, *SD* = 0.78), and lowest in the no MM condition (*M* = 4.50, *SD* = 1.02).

The analysis revealed that NFC was not significant as a covariate, *F*(1, 63) = 2.24, *p* = 0.14, η_p_^2^ = 0.034, indicating NFC did not account for a significant proportion of variance in engagement after controlling for multimedia condition.

The main effect of multimedia condition was significant, *F*(2, 126) = 4.15, *p* = 0.025, η_p_^2^ = 0.062. Pairwise comparisons and Cohen’s d effect size indicated that participants in the rich MM condition reported significantly higher engagement than those in the no MM condition (*p* < 0.001; *d = *0.50) and those in the sparse MM condition (*p* = 0.007; *d* = 0.39). The difference between the sparse and no MM conditions was marginal (*p* = 0.053; *d* = 0.30).

### Time-on-task

The majority of participants spent their time on task. When asked if they walked away from their computer during the study, most participants reported not leaving (89.2%) and some reported leaving for 1–5 min (10.8%). When asked if they navigated to other websites, most participants reported not switching (92.3%), some switched for 1–5 min (6.2%), and one switched for 16+ minutes (1.5%). When asked if they were on another device, most participants reported that they were not (90.8%) and some were for 1–5 min (9.2%).

## Discussion

We aimed to perform a strong test of the multimedia design for learning principles and CTML by designing an experiment that disentangled content and pedagogy from multimedia design. We developed our script independently of our multimedia to ensure one did not depend on the other and we tested the same content using rich, sparse or no multimedia counterbalancing content and multimedia design, using knowledge questions that tested both factual and conceptual learning. Contrary to our hypothesis, the results indicate that multimedia design did not impact performance on a subsequent knowledge test—even after accounting for individual differences in NFC. Conversely, multimedia design significantly impacted the subjective ratings. Our results suggest that, while multimedia design did not directly impact the cognitive aspects of learning, it did appear to significantly impact learners’ subjective experience. Our study is consistent with several studies that demonstrate that subjective ratings do not always align with more objective measures of learning (e.g., Sondermann and Merkt, 2023; Wilson et al., 2018; Yuan et al., 2021). The results are also consistent with CATLM, suggesting that well-designed multimedia will increase motivation and engagement (Moreno, 2005).

By far the most interesting and surprising result was the lack of effect for the multimedia manipulation on participants’ learning. Historically the literature on multimedia design for learning has boasted some of the largest effect sizes in psychology (Mayer, 2002), suggesting that adopting these design features had the potential to have an enormous impact on students’ grades. Although multimedia design principles have been widely validated in laboratory studies, the literature has described relatively few boundary conditions, and many principles have not been systematically tested across diverse learners, materials, or settings. There is even a habit among researchers in this area to default to using a narrow range of content topics, including lightning, brakes and pumps, because they were used in some of the seminal research (Çeken and Taşkın, 2022).

In considering how to interpret this null result, we considered both the strength of our multimedia manipulation and the sensitivity of our measures of learning. While our design was unlikely to perfectly incorporate all the multimedia design principles, we feel that the rich MM condition represents our very best efforts to thoughtfully implement all the relevant empirically supported design principles. Additionally, the no MM condition consisted of a static image of the title slide with no additional visual resources provided to help organize information. After much discussion and consideration among the research team, we genuinely felt that our stimuli represented two ends of the design spectrum and that we could not make our manipulation of multimedia design any stronger.

The knowledge test questions could have lacked sensitivity to distinguish performance either because they were too easy or too difficult. However, our participants were recruited from the general population, not from psychology courses where prior exposure might have made the questions trivial. Moreover, the knowledge test was pilot-tested, and both the pilot data and our experimental data demonstrated appropriate validity and reliability. These findings suggest that the questions were neither uniformly easy nor prohibitively difficult, but rather well-calibrated to detect differences in performance if such differences had been present. Furthermore, the multimedia design condition and topics were fully counterbalanced, and the video order was randomized to control for order effects and ensure comparable difficulty across conditions.

It is possible that the questions in the knowledge test might not require multimedia learning to be answered effectively. However, because the contemporary literature has not provided boundary conditions for content, pedagogy, or learning outcomes, there is no way to operationally define what kinds of content, pedagogy, or learning outcomes might benefit from using the multimedia design principles. Another potential critique is that our study used a combination of factual and transfer questions. However, we wanted our methods to generalize to real post-secondary classrooms, and real assessments are typically a mix of questions that test factual and conceptual knowledge. Moreover, meta-analyses and reviews indicate that effective multimedia design can increase transfer test performance by 20%–75% compared to text-only or poorly designed materials, while gains for factual recall are smaller but still positive (Berney and Detrancout, 2016; Noetel et al., 2022; Xie et al., 2017).

The efforts we have made to standardize our instructional videos and knowledge test questions suggest that our null result is not easily attributed to our methods. Instead, our results may reflect a genuine boundary condition of multimedia learning: when content and pedagogy are clear and conceptually rich, additional visual design may not substantially enhance conceptual understanding.

This interpretation aligns with Clark’s (1994) *Media Equivalence Perspective*, supporting the idea that multimedia design and teaching methods are separate and independent elements of a multimedia-based learning experience. Clark (1994) argued that much of the multimedia learning research confounds multimedia design and teaching methods. It may not be the quality of the materials, but the methods used to teach the material that are responsible for the increase in learning performance. In other words, the use of multimedia design influences the instructor’s teaching approach and vice versa. If an instructor is incorporating design principles into their materials, the teaching will reflect that. The organization of ideas or the use of specific examples are often incorporated into rich multimedia design and would also be reflected in the teaching methods. For example, when an instructor incorporates multimedia elements such as diagrams or signaling cues, this can influence how they pace the lecture, choose examples, or emphasize key points. Conversely, the instructor’s preferred teaching approach can shape the types of multimedia elements they include, resulting in a dynamic, mutually reinforcing relationship between teaching methods and design choices. An instructor’s material tools and immaterial approach interact with each other — they shape and are shaped by one another.

To avoid confounding pedagogy and multimedia design, we were very careful to craft our content scripts separately from designing the multimedia.^2^ Even though the topics were based on sensation and perception and might be expected to benefit from visual teaching aids such as perceptual examples, diagrams, graphs, and organizational graphics, we developed a script that did not refer to any visual elements used in the multimedia. By crafting the content scripts independently of the multimedia design, we ensured that teaching methods and content were experimentally controlled and separable. Under these conditions, the multimedia manipulation had no measurable effect on learning outcomes, confirming that the null result cannot be attributed to confounding between content and design.

While many studies are ambiguous about the development of their learning materials, authors of some prior studies have explicitly described retroactively developing their control conditions by revising their already designed multimedia conditions (see for example Chan et al., 2020; Dousay, 2016; Moreno and Mayer, 2002). Under those conditions, the content has been designed to work in tandem with the multimedia, making it difficult to attribute observed learning effects to either factor alone.

### Instructional equivalence hypothesis

Something that was consistent across all multimedia conditions was the instructor: one person selected the content, wrote the script, and delivered the lecture. Based on our findings, we propose the *Instructional Equivalence Hypothesis*: when teaching methods and content are well-matched and carefully controlled, the presence or richness of multimedia design may have little to no measurable impact on conceptual learning outcomes. While derived from this study, the hypothesis is intended as a generalizable principle, suggesting that the effectiveness of multimedia depends more on the quality of instruction than on the presence of additional visual elements.

Sweller’s et al. (2019) CLT suggests that the role of educators is to help learners manage their cognitive load. The Instructional Equivalence Hypothesis aligns with CLT – both emphasize that learning outcomes depend on how instruction is structured and presented. Our hypothesis makes explicit that multimedia design in particular may add little to learning if cognitive load is already effectively managed through pedagogy.

This interpretation is broadly consistent with Mayer’s (2009) CTML, which emphasizes that multimedia effects should depend on how materials manage cognitive load. However, the Instructional Equivalence Hypothesis runs counter to how the principles of multimedia design for learning are often interpreted in practice. Many researchers treat individual multimedia principles as if they operate in isolation, assuming that adding visuals or distributing information across channels will reliably increase learning. From that perspective, our findings suggest that when instruction is clear and conceptually sufficient, multimedia design may add little to measurable learning outcomes, even if learners perceive it as more engaging. Future tests of the Instructional Equivalence Hypothesis should examine its boundary conditions by varying the type of content, such as comparing STEM topics with topics in humanities. Such studies could determine whether the equivalence of conceptual learning across multimedia conditions holds for different domains, levels of complexity, or types of cognitive processing required.

## Conclusion

Contrary to the predictions of the multimedia design principles for learning, conceptually rich and clearly delivered auditory instruction may be sufficient to support learning without additional multimedia elements. The existing literature on multimedia design for learning is often overly generalized, suggesting that implementing specific design features will produce statistical improvements in learning. This approach neglects the importance of content, pedagogy, and the instructor without actually controlling for those variables.

Our research suggests that the effectiveness of multimedia depends on both instructional methods and design choices, which can interact in ways that either enhance or diminish learning outcomes. Although individual differences among learners may influence outcomes, such variability is expected in any learning environment and does not negate the broader conclusion that clear and coherent instruction can support learning with or without multimedia. Our findings suggest that educators should not feel pressured to become multimedia content creators. In fact, sound pedagogical practice generally avoids dictating the application of uniform design prescriptions. Moreover, there is no singularly correct approach to facilitating learning. Instead, instructors should critically evaluate whether the use of multimedia meaningfully contributes to understanding, effectively communicates the intended content, and justifies the investment of time relative to the significance of the learning objective (Dukewich et al., 2025). Ultimately, the priority remains the clear and accurate communication of content, which does not necessarily require elaborate or complex design.

## Data Availability

The raw data supporting the conclusions of this article will be made available by the authors, without undue reservation.

## Footnotes

^1^We choose to present the knowledge test questions for all videos in a block at the end of the experiment rather than interleafed with the videos to more closely align with what learners experience during their education, which is a mix of content and pedagogical approaches.

^2^This almost certainly constrains our generalizations to educational experiences in which the teaching approach and multimedia materials are developed separately—a scenario that is very unlikely for instructors who employ multimedia in their teaching. However, this approach was necessary to avoid confounding pedagogy and multimedia design.
