# Supplementary material for: When more isn’t better: evidence for an instructional equivalence hypothesis in multimedia design
Source: Front Psychol. 2025 Nov 20;16:1718397. doi: 10.3389/fpsyg.2025.1718397 (PMC12675150; doi:10.3389/fpsyg.2025.1718397)
Supplement: Supplementary file 1 [file Supplementary_file_1.docx]

**Supplementary Materials**

**1. Lecture Engagement Questionnaire**

This questionnaire is extracted from Stull et al. (2018). It is a subjective questionnaire designed to assess a participant’s level of engagement with the lecture material. Questions are answered on a scale of 1, for strongly disagree, to 7, for strongly agree.

| 1.______ | I felt that the subject matter was difficult. |
| --- | --- |
| 2.______ | Please rate the amount of mental effort you put into understanding the material. |
| 3.______ | I enjoyed learning this way. |
| 4.______ | I would like to learn this way in the future. |
| 5.______ | I feel like I have a good understanding of the material. |
| 6.______ | After this lesson, I would be interested in learning more about the material. |
| 7.______ | I found the lesson to be useful to me. |
| 8.______ | I felt like the instructor was working with me to help me understand the material. |
| 9.______ | I found the instructor’s teaching style engaging. |
| 10._____ | I felt motivated to try to understand the material. |
| 11._____ | Please rate the amount of effort you put into understanding the material. |

**2. Need For Cognition Scale**

The following questions are used to evaluate participants’ need for cognition. Participants answer questions on a scale of 1 – 5, 1 being “extremely uncharacteristic” and 5 being “extremely characteristic”. Questions 3-5, 7-9, 12, 16 & 17 are reverse coded.

| 1.______ | I prefer complex to simple problems. |
| --- | --- |
| 2.______ | I like to have the responsibility of handling a situation that requires a lot of thinking. |
| 3.______ | Thinking is not my idea of fun. ** |
| 4.______ | I would rather do something that requires little thought than something that is sure to challenge my thinking abilities. ** |
| 5.______ | I try to anticipate and avoid situations where there is a likely chance I will have to think in depth about something. ** |
| 6. ______ | I find satisfaction in deliberating hard and for long hours. |
| 7.______ | I only think as hard as I have to. ** |
| 8.______ | I prefer to think about small daily projects to long term ones. ** |
| 9.______ | I like tasks that require little thought once I’ve learned them. ** |
| 10._____ | The idea of relying on thought to make my way to the top appeals to me. |
| 11._____ | I really enjoy a task that involves coming up with new solutions to problems. |
| 12._____ | Learning new ways to think doesn’t excite me very much. ** |
| 13._____ | I prefer my life to be filled with puzzles I must solve. |
| 14._____ | The notion of thinking abstractly is appealing to me. |
| 15._____ | I would prefer a task that is intellectual, difficult, and important to one that is somewhat important but does not require much thought. |
| 16._____ | I feel relief rather than satisfaction after completing a task that requires a lot of mental effort. ** |
| 17._____ | It’s enough for me that something gets the job done; I don’t care how or why it works. ** |
| 18._____ | I usually end up deliberating about issues even when they do not affect me personally. |

**3. Time-on-Task Survey/Questionnaire**

This survey was aimed at understanding participants’ time-on-task. Participants were asked multiple questions where they would respond with “for 0 minutes”, “for 1-5 minutes”, “for 6-15 minutes”, and “for 16+ minutes”.

| 1.______ | While the videos were playing, did you walk away from your computer at any point? |
| --- | --- |
| 2.______ | While the videos were playing, did you open another browser window or tab and watch the video picture-in-picture? Ie. Could you still see a small version of the video in the corner of your screen while navigating other websites? |
| 3.______ | While the videos were playing, did you use another device such as a TV, smartphone, or tablet device? |

**4. Table.** *The Principles of Multimedia Design for Learning*

| Origin | Principle | Definition |
| --- | --- | --- |
| Cognitive Theory of   Multimedia  Learning | Multimedia Principle  Modality   Principle | People learn more effectively from words and relevant images compared to words alone.  People learn information more effectively from graphics and spoken narration than from graphics and on-screen text |
|  | Redundancy  Principle | People learn less effectively when identical verbal information is presented as both narration and on-screen text, compared to narration alone. |
|  | Coherence   Principle | People learn more effectively when extraneous material is excluded from multimedia presentations |
|  | Spatial   Contiguity | People learn more effectively when related words and pictures are placed closer together compared to when they are spatially separated. |
|  | Temporal   Contiguity | People learn more effectively when related words and pictures are placed closer together in time compared to when they are temporally separated. |
|  | Signaling Principle | People learn more effectively when cues are provided that point to relevant material or indicate how to organize information. |
|  | Segmenting   Principle | People learn more effectively when material is divided into smaller, more manageable (ideally user-paced) chunks of information. |
|  | Pre-Training Principle | People learn more effectively when they are given definitions and/or the context of a lesson in advance. |
| Cognitive-Affective   Theory of   Multimedia  Learning | Personalization Principle | People learn more effectively from information presented in an informal conversational style rather than in a formal script-based narration. |
|  | Voice   Principle | People learn more effectively from a voice that sounds like a real person rather than a voice that sounds like a machine. |
|  | Embodiment Principle | People learn more effectively with an on-screen agent that uses human gestures (ex. hand movements, facial expressions, body language, etc.). |
|  | Emotional   Design   Principle | This is the proposal that aesthetically pleasing multimedia can improve motivational factors and positive feelings that can have downstream positive effects on learning. |
