# Supplementary material for: When more isn’t better: evidence for an instructional equivalence hypothesis in multimedia design
Source: Front Psychol. 2025 Nov 20;16:1718397. doi: 10.3389/fpsyg.2025.1718397 (PMC12675150; doi:10.3389/fpsyg.2025.1718397)
Supplement: Supplementary file 2 [file Supplementary_file_2.docx]

**Supplementary Materials**

**1. Table A.** *The Principles of Multimedia Design for Learning*

| Origin | Principle | Definition |
| --- | --- | --- |
| Cognitive Theory of   Multimedia  Learning | Multimedia Principle  Modality   Principle | People learn more effectively from words and relevant images compared to words alone.  People learn information more effectively from graphics and spoken narration than from graphics and on-screen text |
|  | Redundancy  Principle | People learn less effectively when identical verbal information is presented as both narration and on-screen text, compared to narration alone. |
|  | Coherence   Principle | People learn more effectively when extraneous material is excluded from multimedia presentations |
|  | Spatial   Contiguity | People learn more effectively when related words and pictures are placed closer together compared to when they are spatially separated. |
|  | Temporal   Contiguity | People learn more effectively when related words and pictures are placed closer together in time compared to when they are temporally separated. |
|  | Signaling Principle | People learn more effectively when cues are provided that point to relevant material or indicate how to organize information. |
|  | Segmenting   Principle | People learn more effectively when material is divided into smaller, more manageable (ideally user-paced) chunks of information. |
|  | Pre-Training Principle | People learn more effectively when they are given definitions and/or the context of a lesson in advance. |
| Cognitive-Affective   Theory of   Multimedia  Learning | Personalization Principle | People learn more effectively from information presented in an informal conversational style rather than in a formal script-based narration. |
|  | Voice   Principle | People learn more effectively from a voice that sounds like a real person rather than a voice that sounds like a machine. |
|  | Embodiment Principle | People learn more effectively with an on-screen agent that uses human gestures (ex. hand movements, facial expressions, body language, etc.). |
|  | Emotional   Design   Principle | This is the proposal that aesthetically pleasing multimedia can improve motivational factors and positive feelings that can have downstream positive effects on learning. |

**2. Table B.** *Pilot Studies Reliability*

| **Pilot Test** | **Sample Size (*N)*** | **Question** | **# of**  **questions** | **Cronbach’s**  **α** | **Notes** |
| --- | --- | --- | --- | --- | --- |
| 1 | 16 | Colour | 14 | .74 | Revised lowest performing questions |
|  |  | Depth | 14 | .75 |  |
| 2 | 30 | Colour | 14 | .76 | Removed four lowest performing questions |
|  |  | Depth | 14 | .77 |  |
| 3 | 25 | Sound | 14 | .67 | Revised & removed three lowest performing |
| 4 | 20 | Sound | 11 | .75 | Removed lowest performing question |

*Note.* At the end of the pilot testing, all three categories (colour, depth, sound) had 10 questions. This made up the final knowledge test used in the full study.

| **3. Table C.** *Demographics Expanded* | | |
| --- | --- | --- |
| **Measure** | **Item** | **Percentage (%)** |
| Language Spoken at Home | English  Other | 16.9%  83.1% |
| Sex | Male  Female | 36.9%  63.1% |
| Education | High School  Bachelor’s Degree  Master’s Degree  Doctoral Degree | 3.1%  67.7%  23.1%  6.2% |
| Educational Preference | Online  In-Person | 76.9%  23.1% |
| Most Common Current Delivery | Online  In-Person  Hybrid  Other | 41.7%  13.3%  36.7%  8.3% |

**4. Lecture Engagement Questionnaire**

This questionnaire is extracted from Stull et al. (2018). It is a subjective questionnaire designed to assess a participant’s level of engagement with the lecture material. Questions are answered on a scale of 1, for strongly disagree, to 7, for strongly agree.

| 1.______ | I felt that the subject matter was difficult. |
| --- | --- |
| 2.______ | Please rate the amount of mental effort you put into understanding the material. |
| 3.______ | I enjoyed learning this way. |
| 4.______ | I would like to learn this way in the future. |
| 5.______ | I feel like I have a good understanding of the material. |
| 6.______ | After this lesson, I would be interested in learning more about the material. |
| 7.______ | I found the lesson to be useful to me. |
| 8.______ | I felt like the instructor was working with me to help me understand the material. |
| 9.______ | I found the instructor’s teaching style engaging. |
| 10._____ | I felt motivated to try to understand the material. |
| 11._____ | Please rate the amount of effort you put into understanding the material. |

**5. Need For Cognition Scale**

The following questions are used to evaluate participants’ need for cognition. Participants answer questions on a scale of 1 – 5, 1 being “extremely uncharacteristic” and 5 being “extremely characteristic”. Questions 3-5, 7-9, 12, 16 & 17 are reverse coded.

| 1.______ | I prefer complex to simple problems. |
| --- | --- |
| 2.______ | I like to have the responsibility of handling a situation that requires a lot of thinking. |
| 3.______ | Thinking is not my idea of fun. ** |
| 4.______ | I would rather do something that requires little thought than something that is sure to challenge my thinking abilities. ** |
| 5.______ | I try to anticipate and avoid situations where there is a likely chance I will have to think in depth about something. ** |
| 6. ______ | I find satisfaction in deliberating hard and for long hours. |
| 7.______ | I only think as hard as I have to. ** |
| 8.______ | I prefer to think about small daily projects to long term ones. ** |
| 9.______ | I like tasks that require little thought once I’ve learned them. ** |
| 10._____ | The idea of relying on thought to make my way to the top appeals to me. |
| 11._____ | I really enjoy a task that involves coming up with new solutions to problems. |
| 12._____ | Learning new ways to think doesn’t excite me very much. ** |
| 13._____ | I prefer my life to be filled with puzzles I must solve. |
| 14._____ | The notion of thinking abstractly is appealing to me. |
| 15._____ | I would prefer a task that is intellectual, difficult, and important to one that is somewhat important but does not require much thought. |
| 16._____ | I feel relief rather than satisfaction after completing a task that requires a lot of mental effort. ** |
| 17._____ | It’s enough for me that something gets the job done; I don’t care how or why it works. ** |
| 18._____ | I usually end up deliberating about issues even when they do not affect me personally. |

**6. Time-on-Task Survey/Questionnaire**

This survey was aimed at understanding participants’ time-on-task. Participants were asked multiple questions where they would respond with “for 0 minutes”, “for 1-5 minutes”, “for 6-15 minutes”, and “for 16+ minutes”.

| 1.______ | While the videos were playing, did you walk away from your computer at any point? |
| --- | --- |
| 2.______ | While the videos were playing, did you open another browser window or tab and watch the video picture-in-picture? Ie. Could you still see a small version of the video in the corner of your screen while navigating other websites? |
| 3.______ | While the videos were playing, did you use another device such as a TV, smartphone, or tablet device? |

**7. Knowledge Test Questions**

**C1** Why does a pumpkin appear orange to our eyes?

(A) Because it reflects the wavelength of light that our visual system interprets as orange

(B) Because it emits the wavelength of light that our visual system interprets as orange

(C) Because it emits orange light

(D) Because it refracts orange light

**C2** Why do evolutionary biologists suggest that primates have developed colour vision?

(A) To find fruit more easily

(B) To detect predators

(C) To recognize prey

(D) To determine when someone is sick

**C3** How might altering the typical colour of an object affect its identification in real-world scenarios?

(A) Because colour is a cue to identity, it could make people slower and less accurate at identification

(B) It would have no effect on identification since shape is the only important factor

(C) Because atypical colours draw attention, it would make people faster and more accurate at identification

(D) It would only affect identification in black and white conditions

**C4** In what ways could the findings from the object identification experiment be applied to improve user interface design?

(A) By ensuring that icons and buttons are in colours that users typically associate with their functions

(B) By using different colours for the icons and buttons to make the interface more vibrant

(C) By avoiding the use of colour for icons and buttons altogether to prevent any confusion

(D) By changing the colours of icons and buttons frequently to keep the interface interesting

**C5** What would a reflectance curve look like for an object that appears black?

(A) It would show very low reflectance across all wavelengths

(B) It would show very high reflectance across all wavelengths

(C) It would show selective reflectance with some wavelengths being reflected more than others

(D) It would show equal reflectance across all wavelengths

**C6** What type of photopigments do rods contain, and how does it affect their function?

(A) Rods contain one type of photopigment, making them colourblind but highly sensitive to light

(B) Rods contain three types of photopigments, allowing them to detect colour

(C) Rods contain no photopigments, making them insensitive to light

(D) Rods contain multiple photopigments, making them sensitive to all wavelengths of light

**C7** How can the concept of achromatic and chromatic colours be applied to improve the design of visual aids for educational purposes?

(A) By using achromatic colours to reduce distractions and chromatic colours to highlight important information

(B) By using only achromatic colours for all information

(C) By avoiding the use of colour in visual aids

(D) By using random colours to make visual aids more interesting

**C8** How might the understanding of the role of photoreceptors in colour perception be applied to create more effective visual signals?

(A) By designing signals that use chromatic hues corresponding to the peak sensitivities of cones

(B) By designing signals that use achromatic hues corresponding to the peak sensitivities of cones

(C) By designing signals that use chromatic hues corresponding to the peak sensitivities of rods

(D) By designing signals that use achromatic hues corresponding to the peak sensitivities of rods

**C9** In the video, it is mentioned that two squares are physically the same colour but appear different. How can this phenomenon be explained?

(A) By the context and surrounding colours influencing our perception

(B) By the inherent colour properties of the squares changing

(C) By the light source changing the colour of the squares

(D) By the squares emitting different wavelengths of light

**C10** Colour serves as a signalling function. Which of the following is an example of this function?

(A) Identifying ripe fruit from unripe fruit

(B) Picking out colourful fish from a blue ocean background

(C) Recognizing a firetruck by its bright red colour

(D) Choosing a vibrant colour for a sports car to make it stand out

**D1** Which of the following is NOT an advantage of having two eyes?

(A) It makes it easier for animals to see colours

(B) If an animal experiences an injury to one eye they will still be able to see

(C) It allows animals to see more of their visual environment at one time

(D) It makes it easier for animals to spot small, fast-moving objects

**D2** What is the significance of seeing different images with each eye?

(A) It enhances depth perception and spatial understanding

(B) It helps in detecting colour differences

(C) It increases the visual field

(D) It makes movement more apparent

**D3** In the example of the electricity poles, why do we perceive one pole as being further away?

(A) Because it takes up less space in our visual field

(B) Because it is physically smaller

(C) Because it appears smaller

(D) Because it is moving slower

**D4** How does the understanding of relative size help in creating realistic animations?

(A) By ensuring distant objects take up less space on the screen

(B) By making distant objects appear larger

(C) By changing the colour of distant objects

(D) By increasing the brightness of distant objects

**D5** What is the difference between relative size and familiar size as depth cues?

(A) Relative size assumes objects are the same size, while familiar size uses knowledge of an object's typical size

(B) Relative size is used for common objects, while familiar size is used for novel objects

(C) Relative size is based on shape, while familiar size is based on location

(D) Relative size is used in artificial environments, while familiar size is used in natural environments

**D6** What is the potential limitation of using familiar size as a depth cue?

(A) It can lead to incorrect interpretations based on assumptions

(B) It only provides information about relative distance

(C) It only works in brightly lit environments

(D) It only provides information about relative order of objects

**D7** In which of the following scenarios would motion parallax be most noticeable?

(A) As a passenger on a train watching the countryside

(B) In a first-person video game, playing a character walking through an abandoned village

(C) Seated on a bench as crowds of people on the street walk past you

(D) Watching TV in your apartment, with various scene cuts and camera moves to help create a sense of depth

**D8** How might an architect use the principles of depth perception to design a building that appears taller than it actually is?

(A) Exploiting linear perspective by making the building's lines converge at a higher point

(B) Exploiting atmospheric perspective by painting the building a hazy blue

(C) Exploiting relative size by placing a large sculpture outside the building

(D) Exploiting occlusion by placing a billboard in front of the building

**D9** How might a painter create a sense of depth in a landscape painting?

(A) By making distant mountains appear hazier and bluer

(B) By using bright, saturated colours for distant objects

(C) By ensuring all elements are equally sharp and clear

(D) By making the horizon line lower and more prominent

**D10** How can the principle of texture gradient be used in interior design to make a room appear larger?

(A) By using patterns that become more closely packed as they recede into the distance

(B) By using large, uniform patterns throughout the room

(C) By avoiding any patterns or textures

(D) By using bright, solid colour

**S1** A frequency spectrum shows a tone that is composed of frequencies of 440Hz, 880Hz, and 1320Hz. The 440Hz frequency is the ______, and the 880Hz and 1320Hz frequencies are both called _____ in this example.

(A) Fundamental frequency; harmonics

(B) Fundamental frequency; secondaries

(C) First harmonic; fundamental frequencies

(D) Primary harmonic; fundamental frequencies

**S2** Why is 0 dB considered the threshold of hearing for humans?

(A) It's the average threshold for young, healthy listeners

(B) It represents an event with no sound

(C) It corresponds to the average pain threshold

(D) It reflects the absence of air movement during sound production

**S3** Which scenario best exemplifies the importance of timbre?

(A) Two singers holding the same note, sounding different

(B) Two notes at different volumes

(C) A saxophone playing a note at different pitches

(D) A tone increasing in loudness

**S4** Why can a flute produce a tone closer to a pure tone than most other instruments?

(A) It uses fewer harmonics

(B) It lacks a fundamental frequency

(C) Its sine wave is non-repeating

(D) It vibrates through air rather than strings

**S5** Which auditory quality is most closely linked to the frequency of a sound wave?

(A) Pitch

(B) Timbre

(C) Loudness

(D) Amplitude

**S6** How does a harmonic spectrum relate to musical pitch?

(A) The fundamental frequency defines the pitch

(B) It distorts frequency perception

(C) The presence of harmonics masks the pitch

(D) All harmonics must be identical in amplitude

**S7** What kind of sound would a spectrum with equal intensity across all audible frequencies represent?

(A) White noise

(B) Ambient noise

(C) An inharmonic tone

(D) A complex tone

**S8** In the context of sound waves, what does the region of condensation represent?

(A) An area where air molecules are tightly packed, resulting in high pressure

(B) An area where air molecules are pulled apart, resulting in low pressure

(C) A decrease in the amplitude of the wave

(D) The gradual fading of sound intensity during decay

**S9** How does Fourier analysis help explain the difference in sound quality (timbre) between two instruments playing the same note?

(A) By revealing differences in harmonic structure and amplitude

(B) By identifying the variations in loudness

(C) By showing differences in their fundamental frequencies

(D) By isolating the sound’s attack and decay

**S10** What role does the "attack" of a sound play in auditory perception?

(A) It shapes the initial rise in sound energy, helping us recognize the source

(B) It affects the speed at which sound waves travel

(C) It influences how quickly the sound fades after it ends

(D) It reflects changes in pitch over time

**8. Counterbalancing & Randomization**

Multimedia format (rich, sparse, none) was fully counterbalanced across three content topics (color, depth, sound). This ensured that content was orthogonal to multimedia design, eliminating potential confounds between content, pedagogy, and format. Participants were randomly assigned to one of the following 6 conditions:

| **VIDEO A** | **VIDEO B** | **VIDEO C** |
| --- | --- | --- |
| rich-color | sparse - depth | no - sound |
| rich - color | sparse - sound | no - depth |
| rich - depth | sparse - color | no - sound |
| rich - depth | sparse - sound | no - color |
| rich - sound | sparse - color | no - depth |
| rich - sound | sparse - depth | no -color |

The order of the video conditions (ie. Video A, B, and C) were presented randomly to account for order effects.

**9. Results Tables**

**Table D.** *ANOVA Results for Knowledge Test*

|  | F | Sig. | Partial Eta Squared |
| --- | --- | --- | --- |
| NFC | 0.25 | .62 | .004 |
| Multimedia | 0.52 | .60 | .008 |
| Multimedia*NFC | 0.78 | .46 | .012 |

**Table E.** *Bayesian Repeated Measures ANOVA Results*

| Models | P(M\|data) | BF_M_ | BF_01_ | Error % |
| --- | --- | --- | --- | --- |
| NFC | 0.26 | 1.04 | 2.28 | 2.76 |
| Multimedia | 0.11 | 0.35 | 5.57 | 0.67 |
| Multimedia + NFC | 0.05 | 0.15 | 12.3 | 4.31 |

**Table F.** *ANOVA Results for Subjective Ratings*

|  | F | Sig. | Partial Eta Squared |
| --- | --- | --- | --- |
| NFC | 2.24 | .14 | .034 |
| Multimedia | 4.15 | .025 | .062 |
| Multimedia*NFC | 2.43 | .092 | .037 |
